# Supplementary material for: RNANetMotif: Identifying sequence-structure RNA network motifs in RNA-protein binding sites
Source: PLoS Comput Biol. 2022 Jul 12;18(7):e1010293. doi: 10.1371/journal.pcbi.1010293 (PMC9275694; doi:10.1371/journal.pcbi.1010293)
Supplement: S1 Method — A. Selection of control sites for the evaluation of network modules. B. Summary of parameters in RNANetMotif algorithm. C. Parameters in RNA secondary structure prediction. (DOCX) [file pcbi.1010293.s003.docx]

1. **Selection of control sites for the evaluation of network modules**
2. **Summary of parameters in RNANetMotif algorithm**
3. **Parameters in RNA secondary structure prediction**

**A. Selection of control sites for the evaluation of network modules**

To evaluate the significance of network modules of a specific RBP, we selected negative sites from non-target RBP binding sites' sets as a control set. To ensure that the negative set of each RBP has similar nucleotide frequencies as the corresponding positive set, we adopted a greedy strategy. The detailed steps are as follows.

Denote the target RBP as $P$, other 21 RBPs as $\mathrm{OP}$. And the set containing the binding sites of $P$ is denoted as $S_{P}$, binding sites of $\mathrm{OP}$ as $S_{OP}$. Each 100nt site $s$ in $S_{P}$ and $S_{OP}$ is mapped to a 4D vector $v_{s}=(v_{sA}, v_{sC},v_{sG},v_{sU})$, where $v_{st}, t\in\left\{ A,C,G,U \right\}$ is the frequency of $t$ in $s$. And A%, C%, G%, U% distribution in binding sites of every RBP is displayed in **Supporting Fig S6**.

Define the Chebyshev distance between two sites $s_{i}$ and $s_{j}$ as

$$D_{Chebyshev}\left( s_{i}, s_{j} \right)=D_{Chebyshev}\left( v_{s_{i}}, v_{s_{j}} \right)={max}_{n\in\left\{ A,C,G,U \right\}}(|v_{s_{i}n}-v_{s_{j}n}|)$$

| **Algorithm 1.** Greedy algorithm |
| --- |
| 1: $\mathbf{procedure} G\mathrm{REEDY} (S_{P}, S_{\mathrm{OP}})$ |
| 2: $\mathrm{index}\leftarrow0$ |
| 3: $N\leftarrow\{ \}$ |
| 4: **while** \|N\|<\|$S_{P}$\| **do** |
| 5: $next\_neg\leftarrow{argmin}_{c\in S_{OP}\backslash N}D_{Chebyshev}\left( S_{P}\left( index \right), c \right)$ |
| 6: $N\leftarrow N\cup\{next\_neg\}$ |
| 7: $index\leftarrow index+1$ |
| 8: **end while** |
| 9: $p\_value\leftarrow KS\_test(S_{P}, N)$ |
| 10: **if** p_value ≤ ε **then** |
| 11: **return** N |
| 12: **end if** |
| 13: $\mathbf{end procedure}$ |

We then removed redundant and missing data for control sets of each RBP using module 'cd-hit-est' from CD-HIT at 80% similarity. For each RBP, negative sites with sequence similarity above 80% to any positive sites were excluded using module 'cd-hit-est-2d' from the CD-HIT tool.

**B. Summary of parameters in RNANetMotif**

In RNANetMotif algorithm, there are parameters in each step. We conclude and list all parameters as follows:

In Methods 4.2:

$$RNAplfold (W, L, u)$$

Default: $W$ =100, $L$ =100, $u$ =1

In Methods 4.4:

$$GraphK (k, compactness score cutoff)$$

Default: $k$ =3 or 4 or 5, $compactness score cutoff=1/(2\times(k-1)+0.5$)

In Methods 4.5:

1. $HVDM (\#classes)$
2. $Similarity network construction \left( n, w_{cutoff} \right)$

Default: $\#classes$=4, $n=2\%\times size of V_{S}, w_{cutoff}=0.9 quantile of all weights$

In Methods 4.6:

1. $ClusterONE (s, d,max\_overlap)$
2. $Network motif mining process (maximal clique size cutoff, number of EKS motifs)$

Default: $s=100, d=0.8,max\_overlap=0.2 ,$ $maximal clique size cutoff$= top 50%, $number of EKS motifs$= top 50

**C. Parameters in RNA secondary structure prediction**

We experiment on different parameters of RNAplfold. RNAplfold has two important parameters: the size of the window (W) and the maximum base pair span (L). Munteanu et al. have proved that the prediction of RNAplfold has a clear trend of more base pairs as the W and/or L increase, but the optimal parameter values still be unclear [1]. Many researches use RNAplfold with the values (W, L) = (80, 40) [2-4], while Lange et al. recommend that W = L + 50 in order to have each base present in at least 51 windows [5]. The current default parameter setting of RNAplfold is W=L=70. We test on four (W, L) values in [(100, 100), (100, 50), (80, 40), (70, 70)] and used RNANetMotif as a supervised classifier to report the robust performance on different (W, L) values (see **Supporting Fig S11**).

In this study we used RNAplfold to predict RNA base pairing probabilities. We considered several other RNA secondary structure prediction methods, RNAfold, RNAaliford, Rfold, and RNAstructure.

RNAfold and RNAaliford are both parts of the Vienna package: RNAfold is tailored towards global secondary structure prediction while RNAaliford is aimed at predicting from an alignment of evolutionarily related RNA sequences. Since we are studying local RNA structure motifs, neither of these methods is optimal for our purpose. We note that RNAplfold is widely used in many other RBP-RNA studies, e.g., RNAcontext, RNAcommender, RCK.

To explore the accordance between different RNA structure prediction tools, we compared the results of RNAplfold, RNAstructure, RNAfold and calculated the Jaccard similarity of the predicted paired nodes set and base pair set. The results are show in **Supporting Fig S12**, showing general consistency among these three software tools. We have added a function in webserver that user can provide and upload their bp files to our tool.

**Reference**

1. Munteanu, A., N. Mukherjee, and U. Ohler, *SSMART: sequence-structure motif identification for RNA-binding proteins.* 2018. **34**(23): p. 3990-3998.

2. Marín, R.M. and J. Vaníček, *Efficient use of accessibility in microRNA target prediction.* 2011. **39**(1): p. 19-29.

3. Li, X., et al., *Predicting in vivo binding sites of RNA-binding proteins using mRNA secondary structure.* 2010. **16**(6): p. 1096-1107.

4. Kazan, H., et al., *RNAcontext: a new method for learning the sequence and structure binding preferences of RNA-binding proteins.* 2010. **6**(7): p. e1000832.

5. Lange, S.J., et al., *Global or local? Predicting secondary structure and accessibility in mRNAs.* Nucleic Acids Res, 2012. **40**(12): p. 5215-26.
