# Supplementary figures and images for: RNANetMotif: Identifying sequence-structure RNA network motifs in RNA-protein binding sites
Source: PLoS Comput Biol. 2022 Jul 12;18(7):e1010293. doi: 10.1371/journal.pcbi.1010293 (PMC9275694; doi:10.1371/journal.pcbi.1010293)

k=3

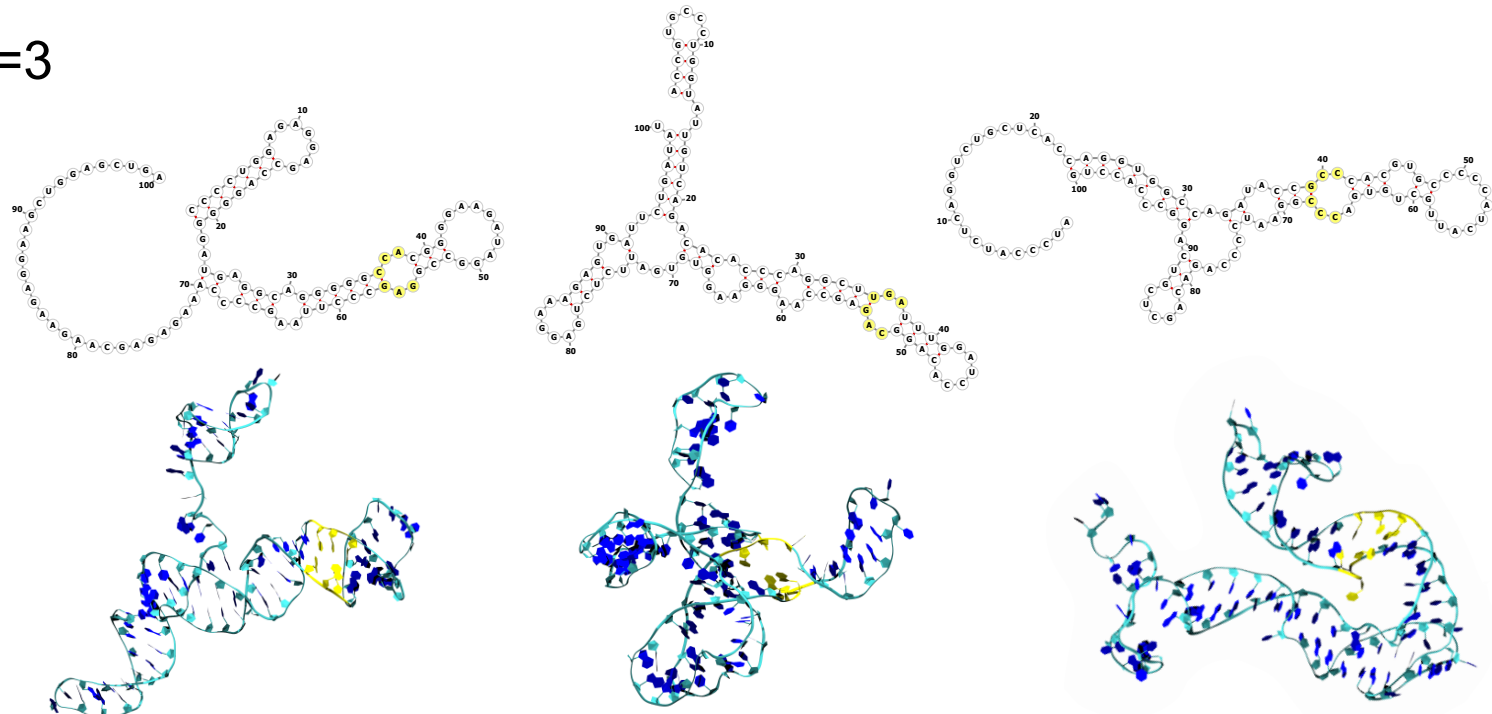

k=4

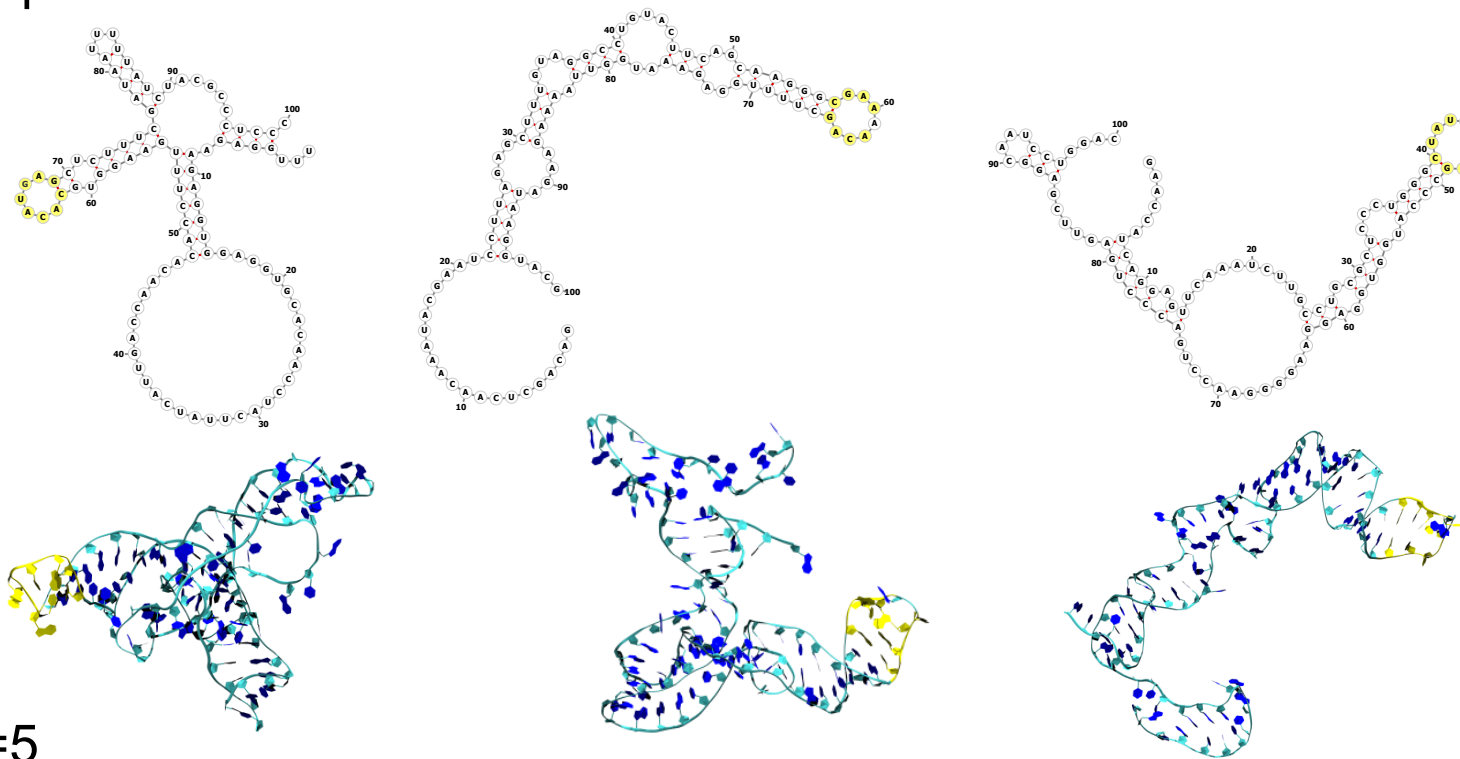

k=5

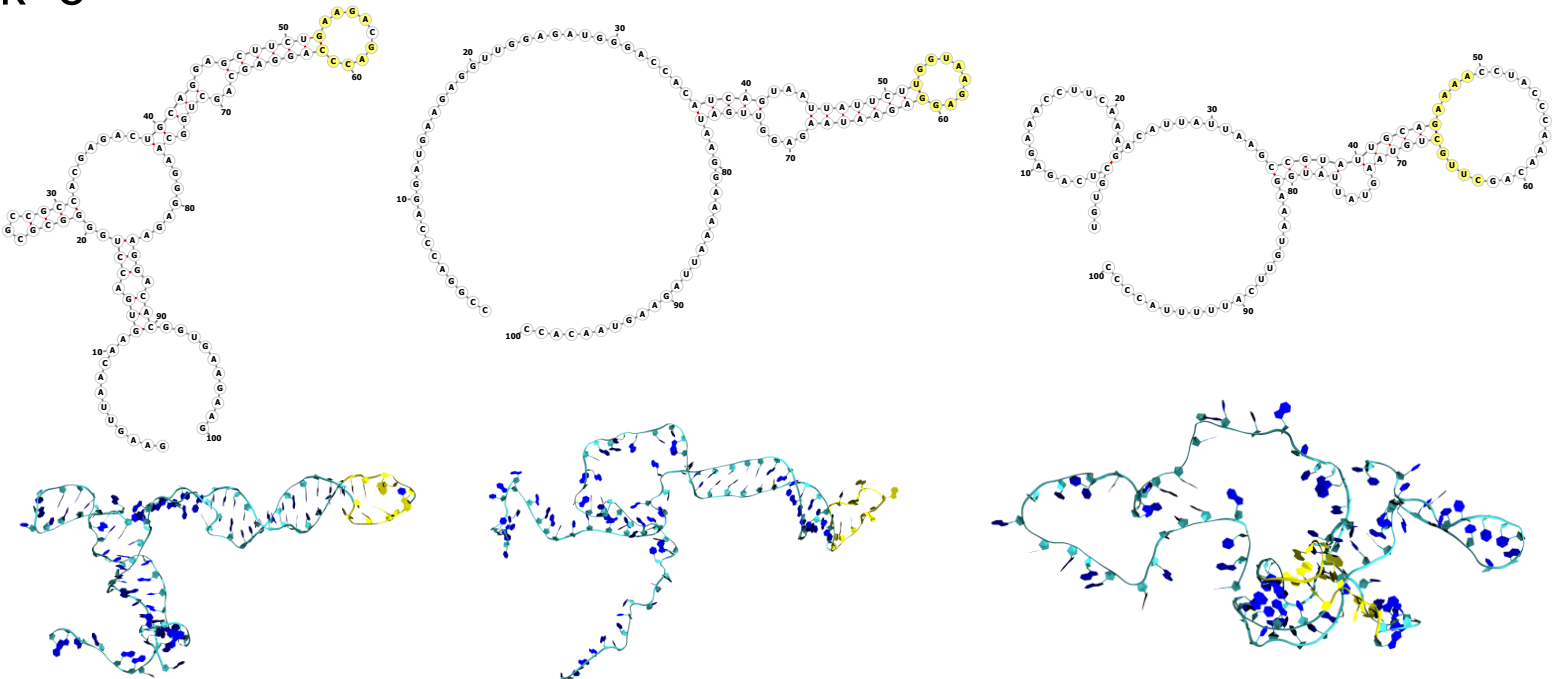

Supplement: S1 Fig — (PDF) [file pcbi.1010293.s004.pdf]

k=3

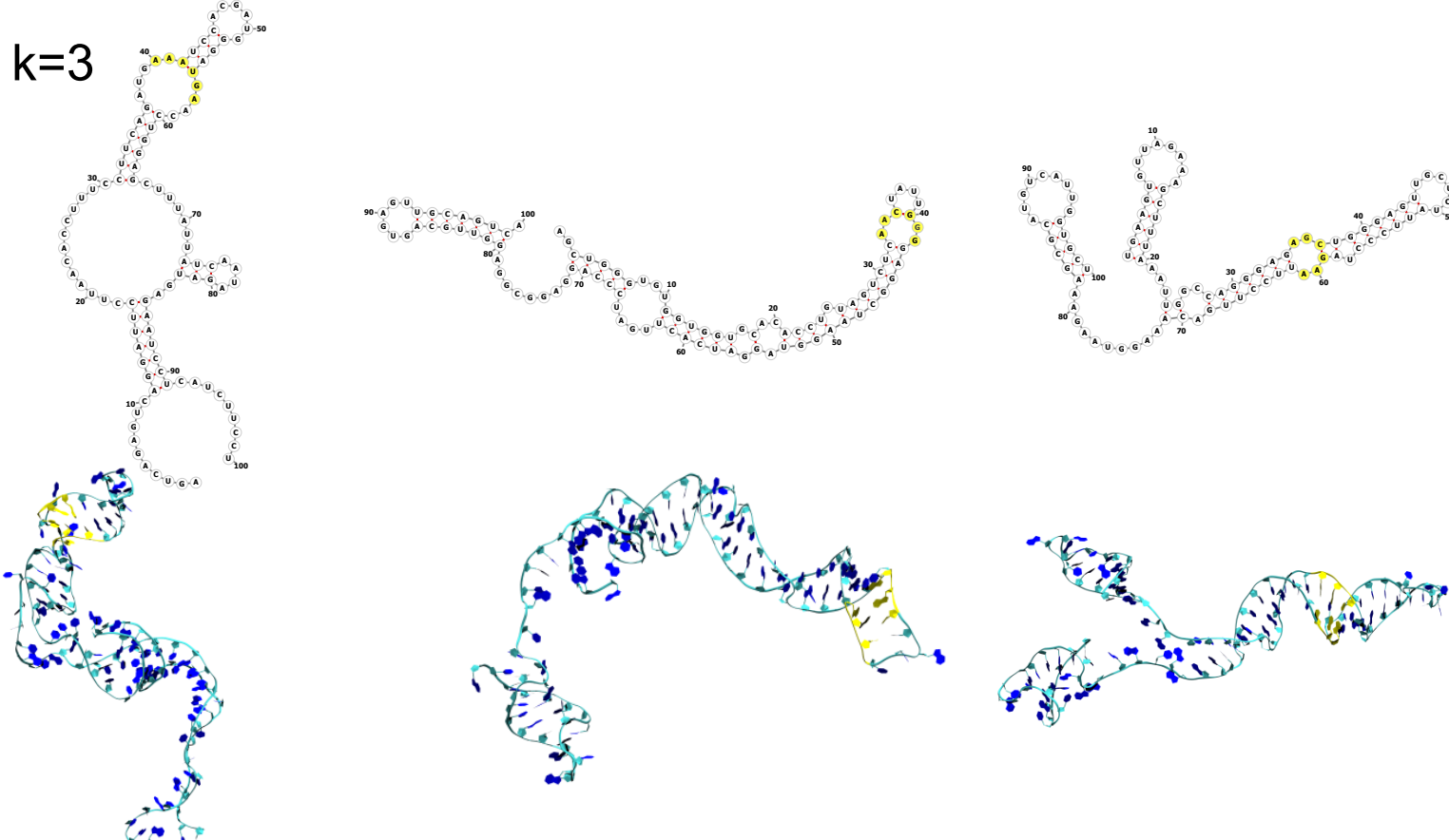

k=4

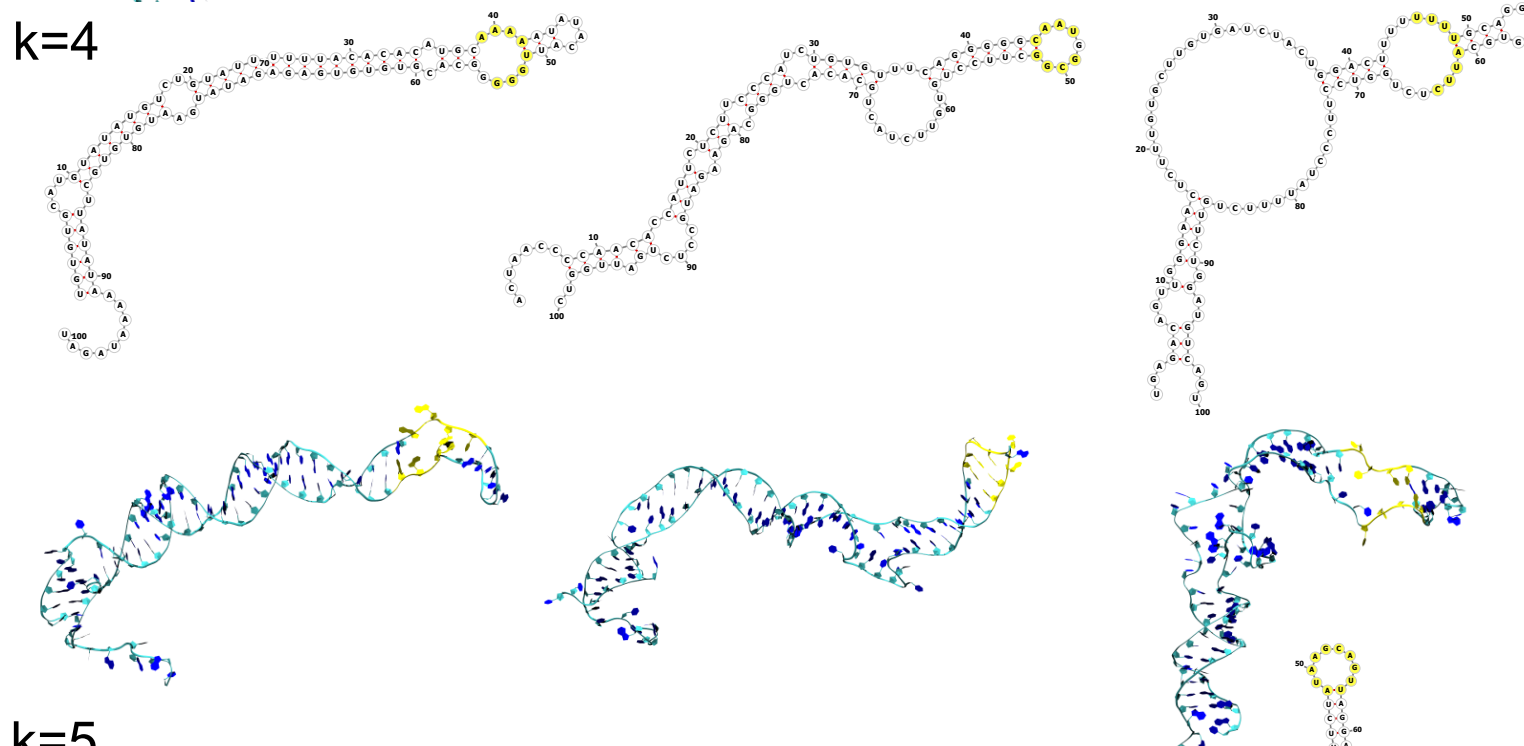

k=5

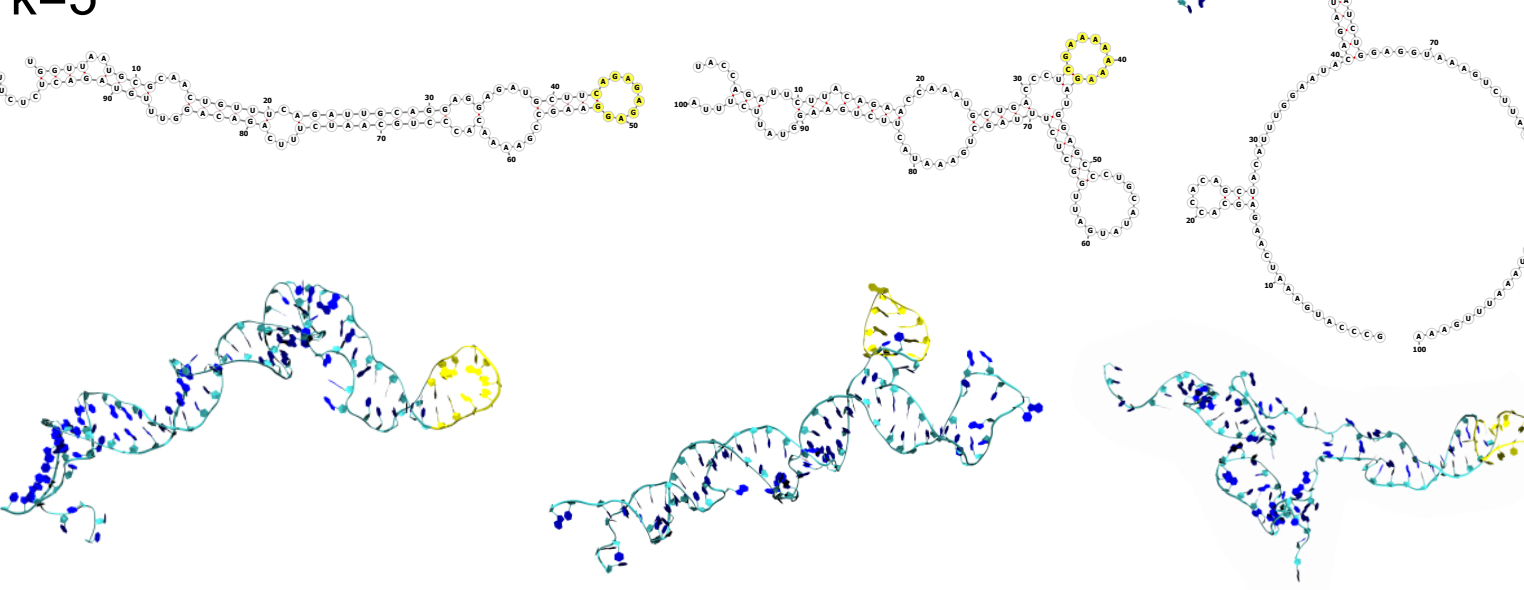

Supplement: S2 Fig — (PDF) [file pcbi.1010293.s005.pdf]

k=3

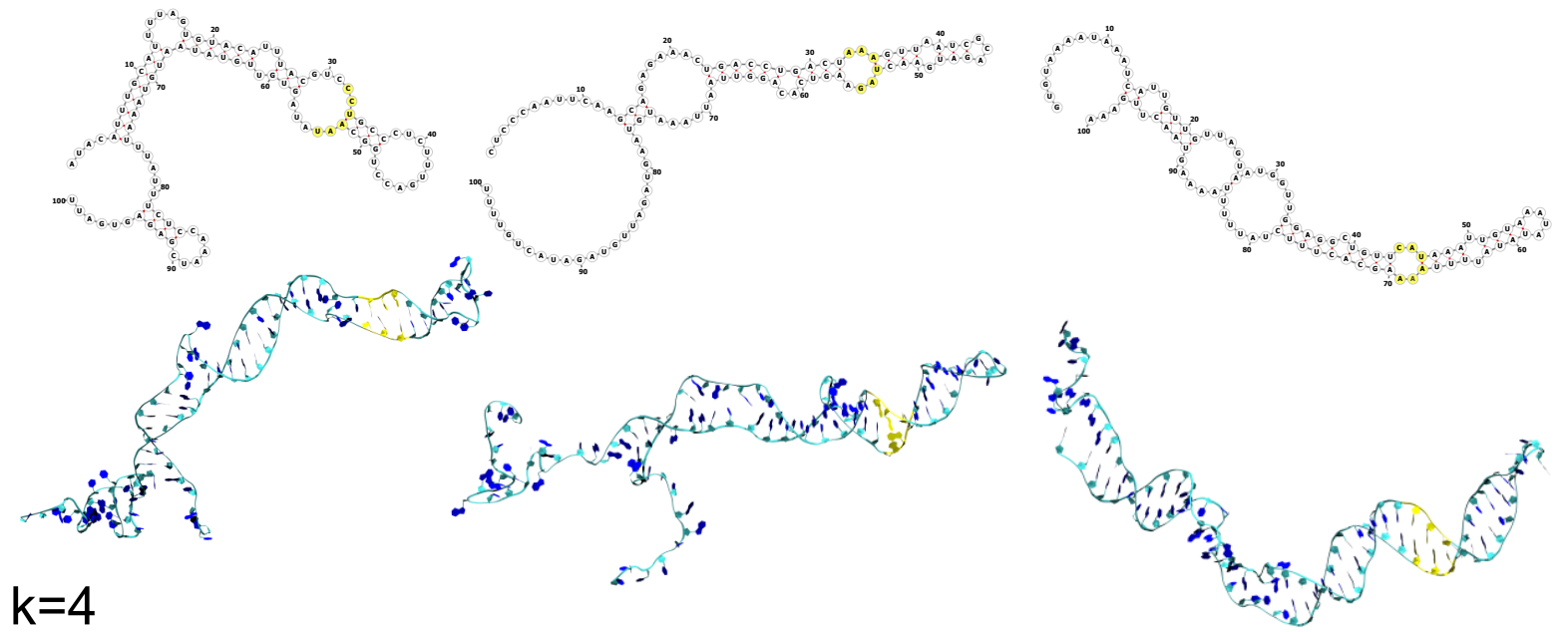

k=4

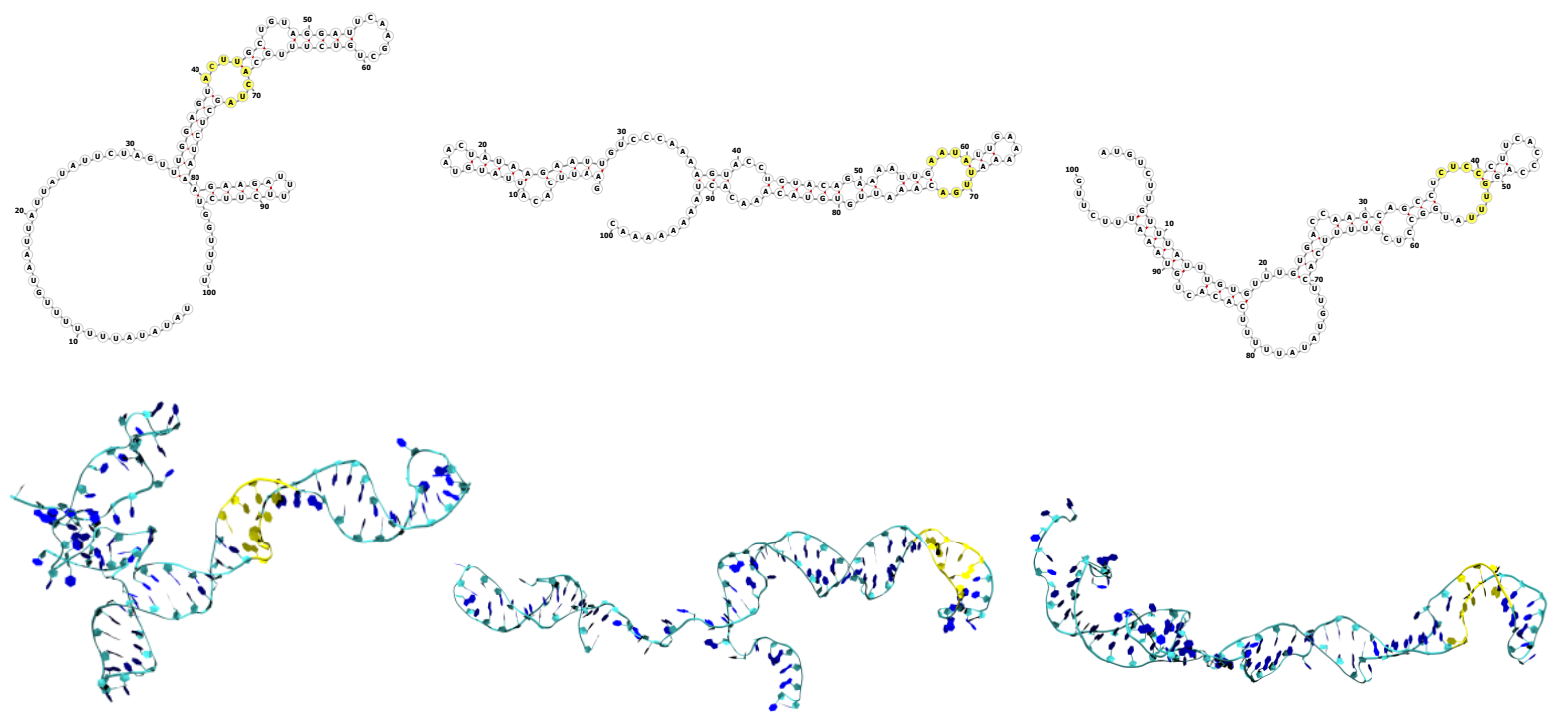

k=5

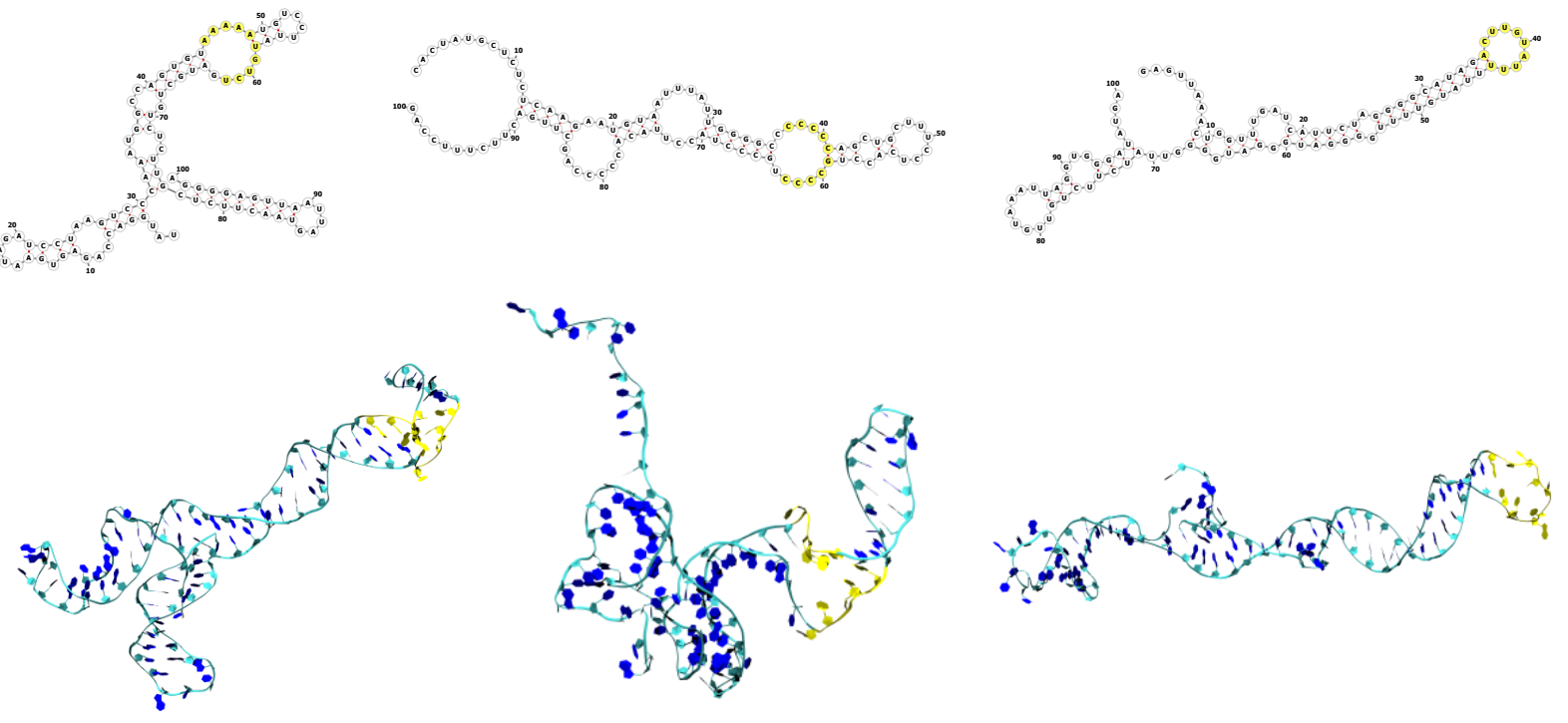

Supplement: S3 Fig — (PDF) [file pcbi.1010293.s006.pdf]

k=3

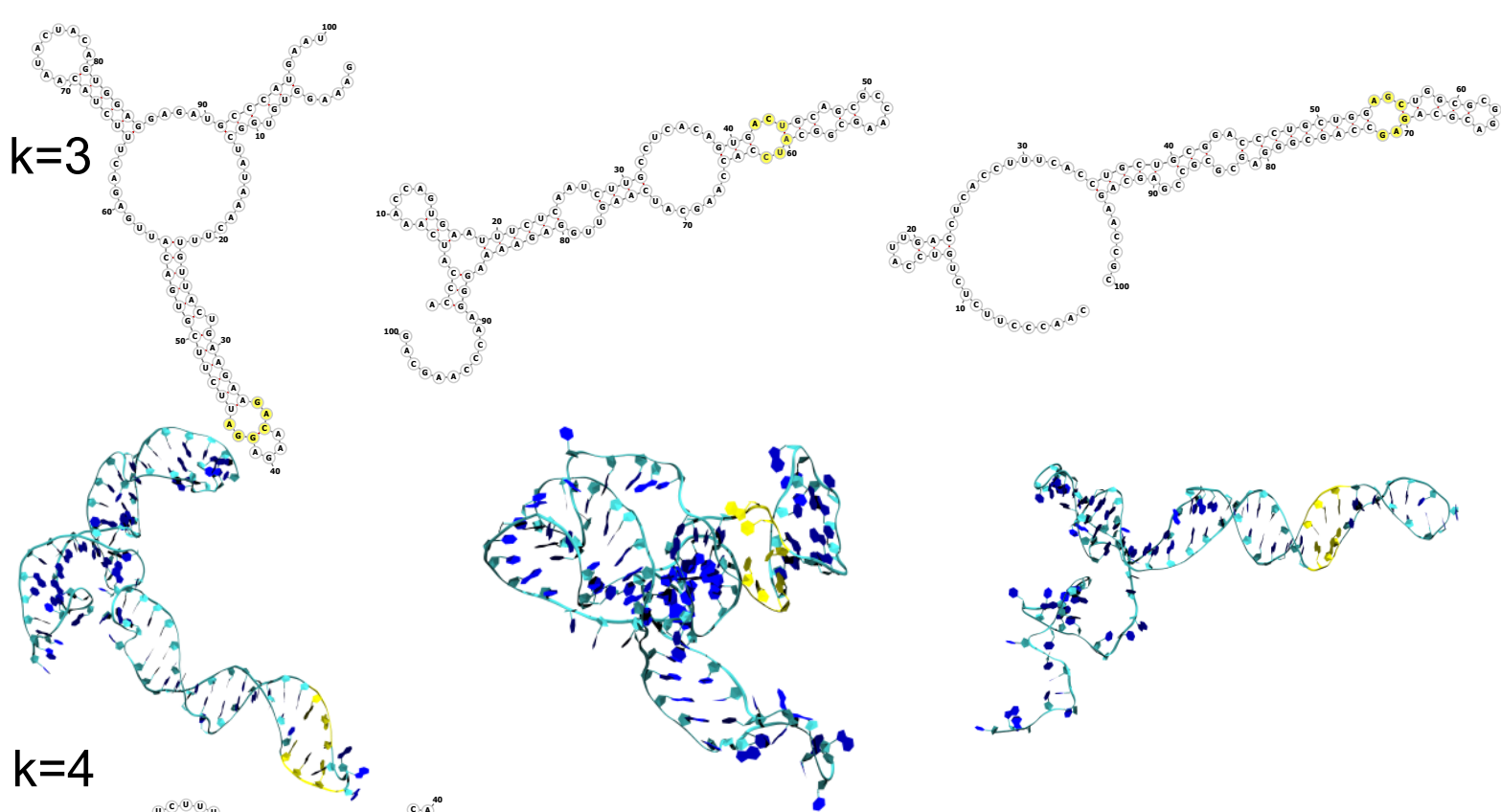

k=4

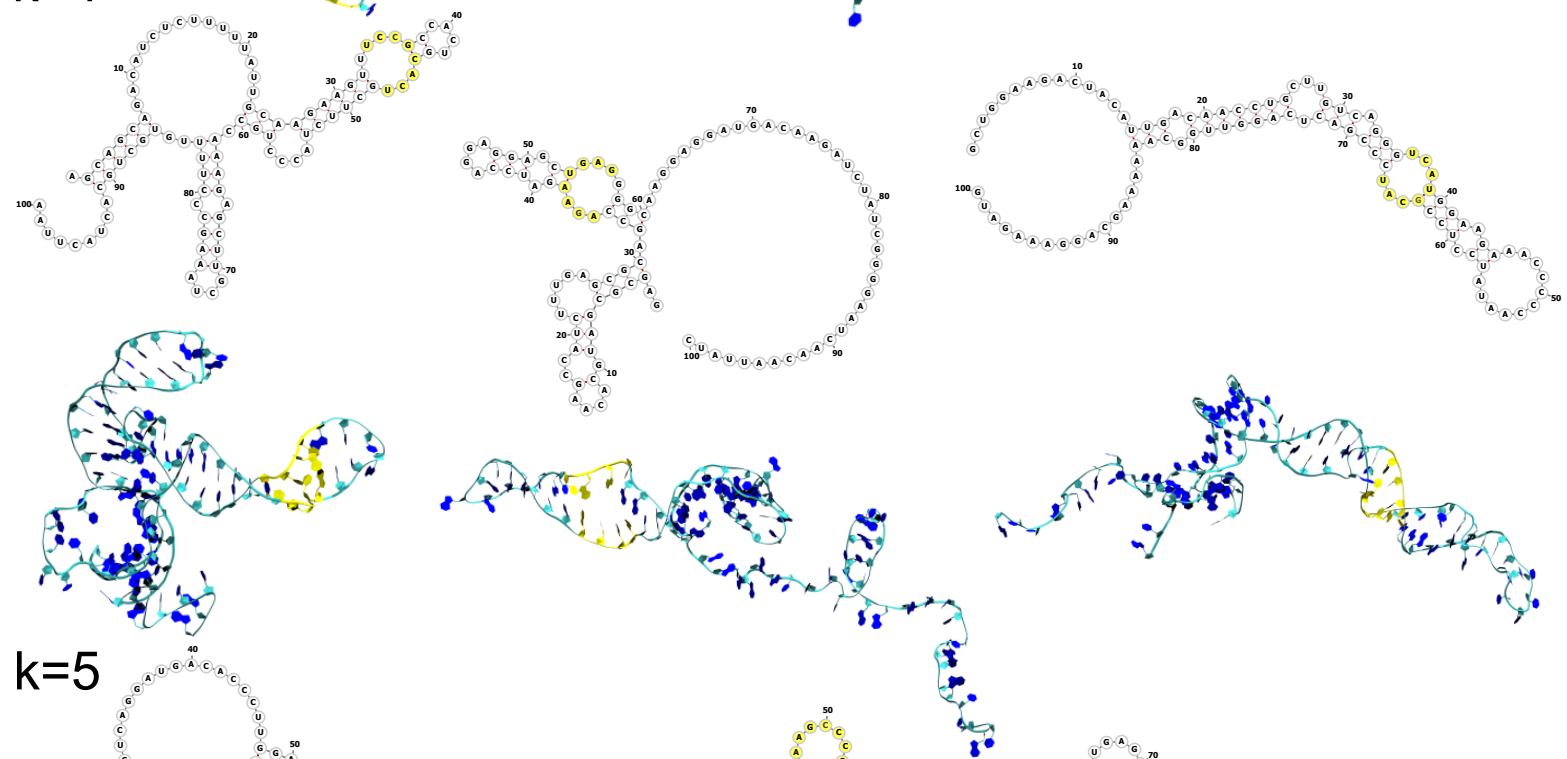

k=5

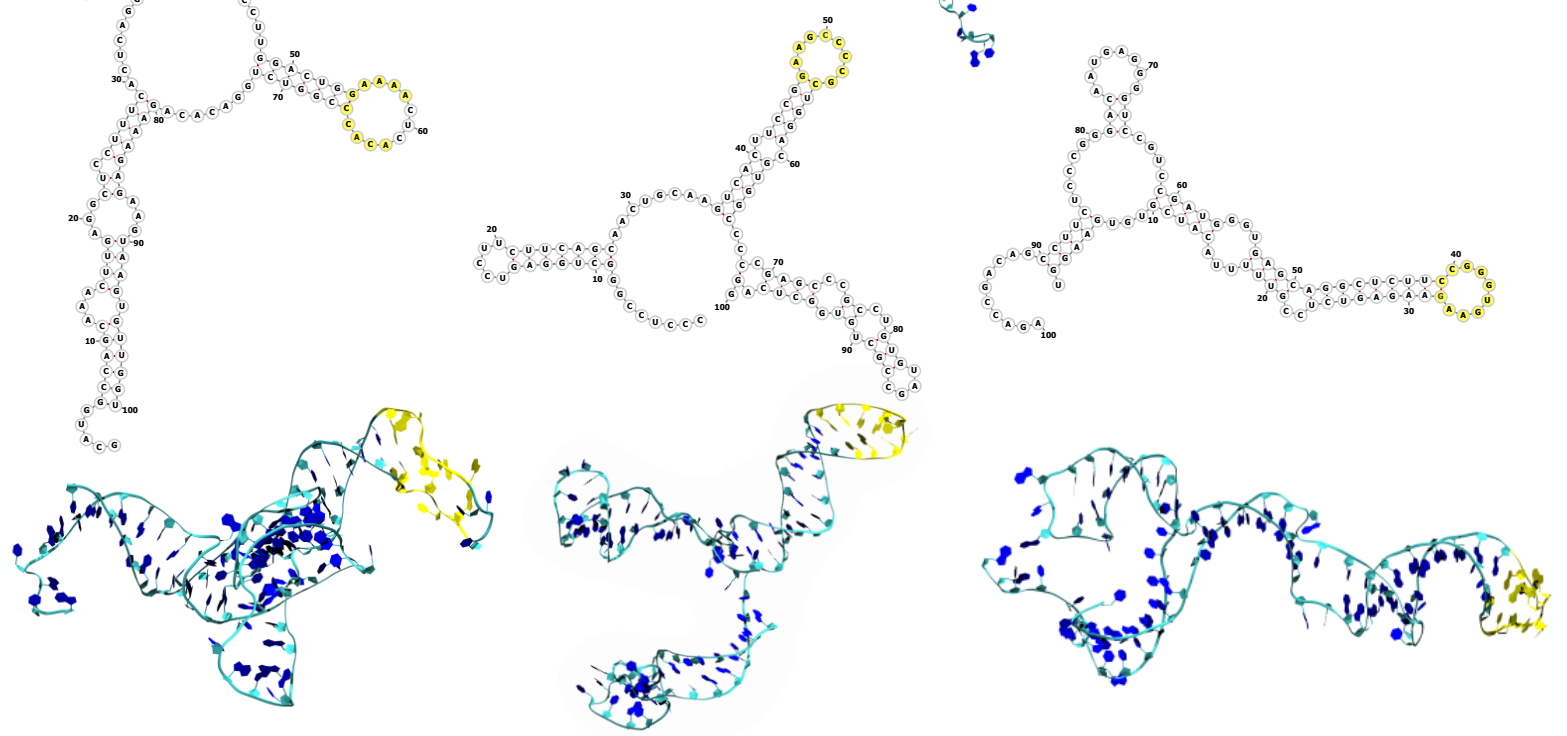

Supplement: S4 Fig — (PDF) [file pcbi.1010293.s007.pdf]

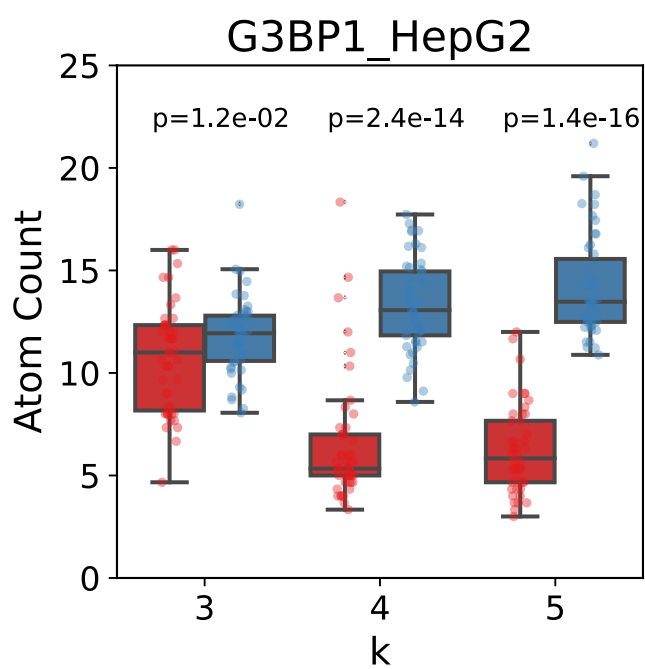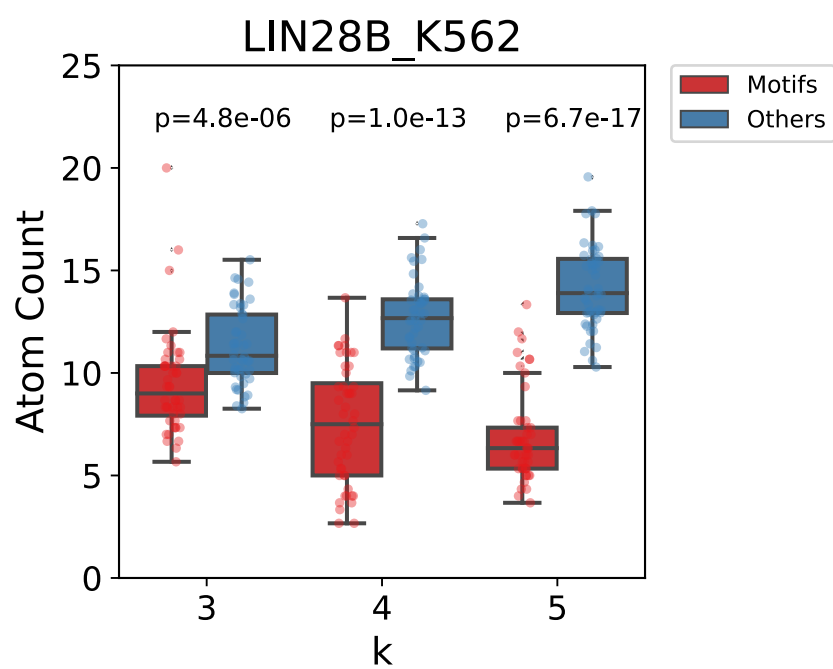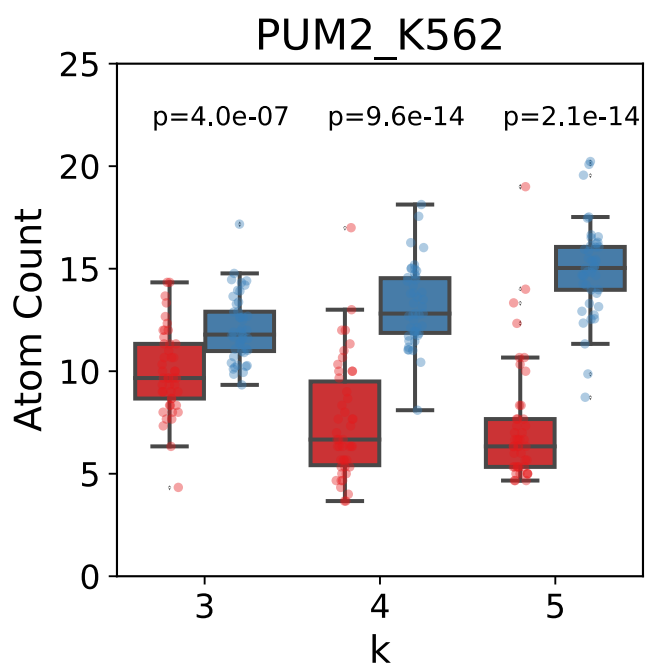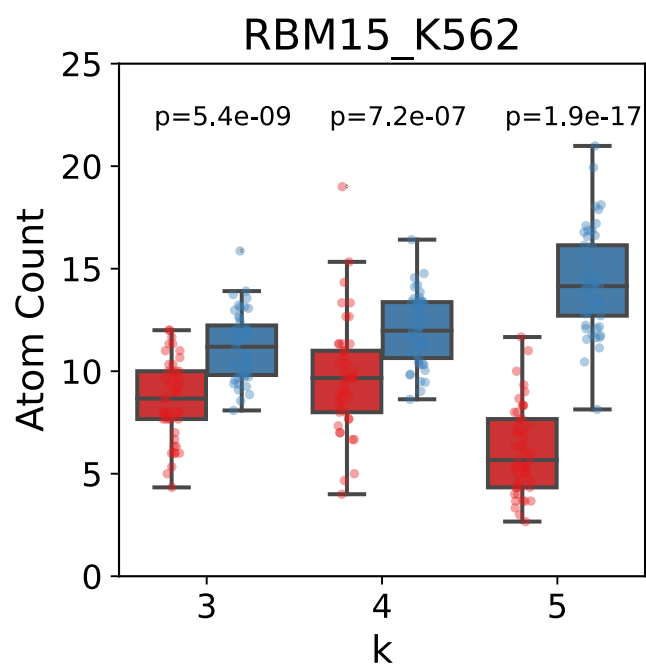

Supplement: S5 Fig — (PDF) [file pcbi.1010293.s008.pdf]

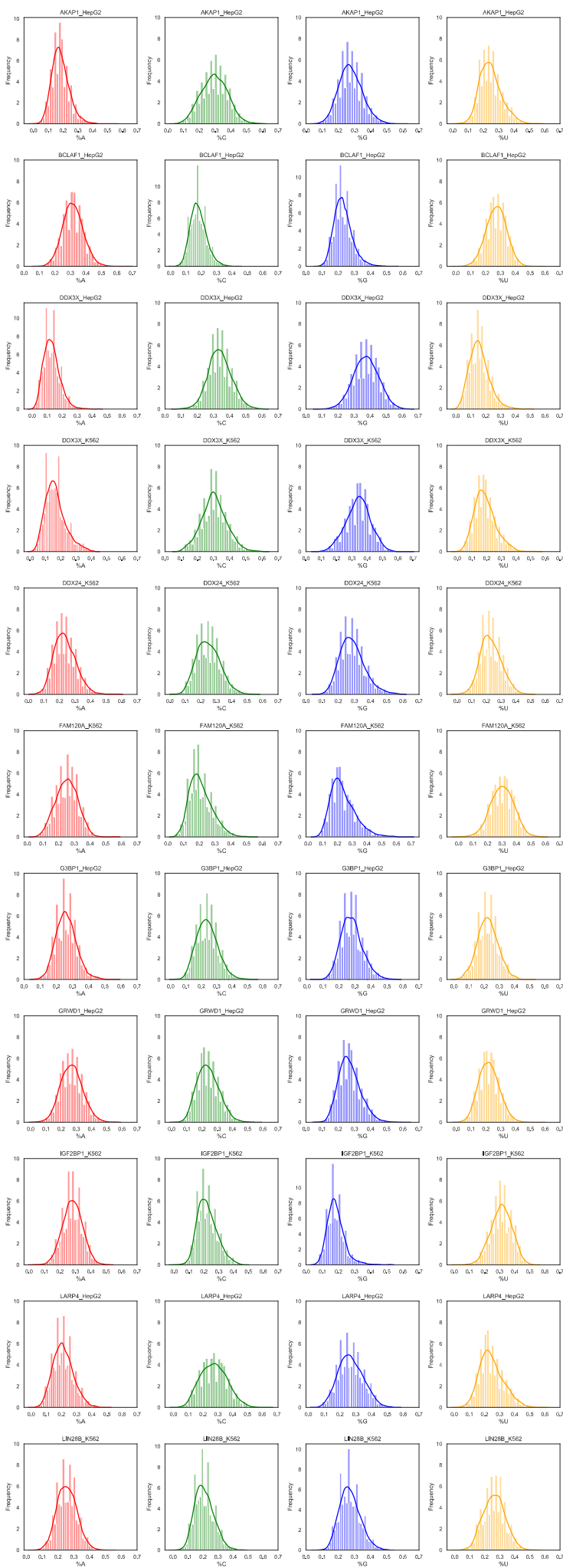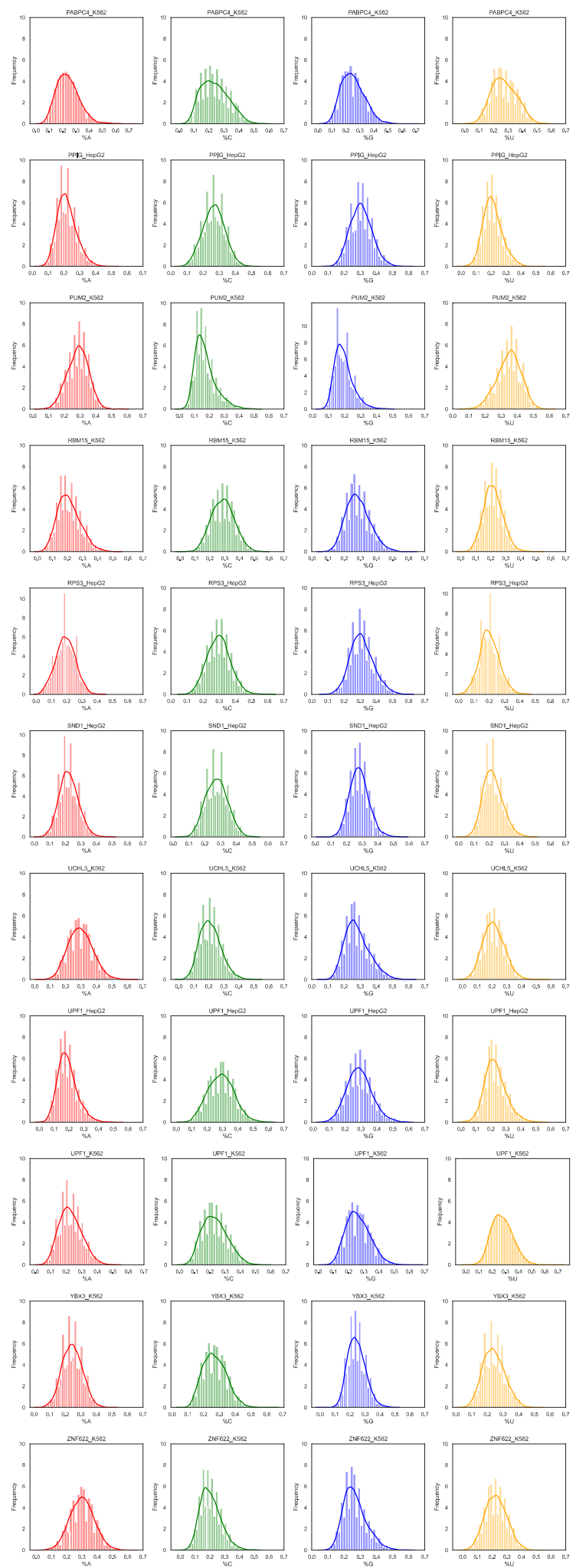

Supplement: S6 Fig — (PDF) [file pcbi.1010293.s009.pdf]

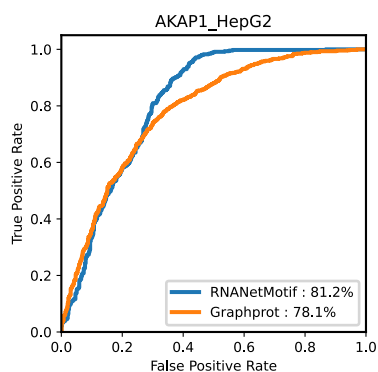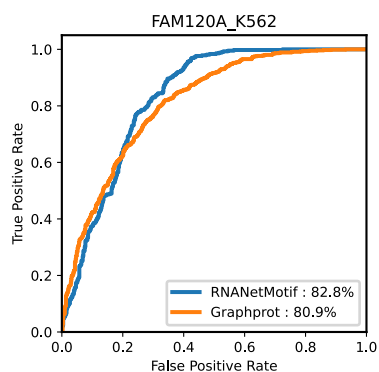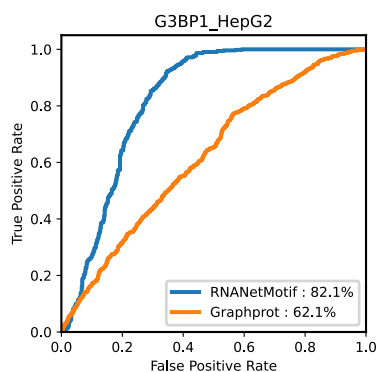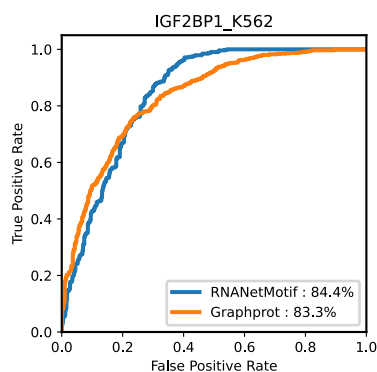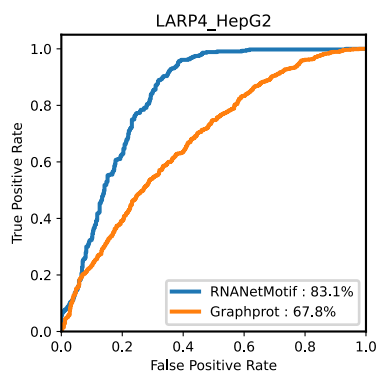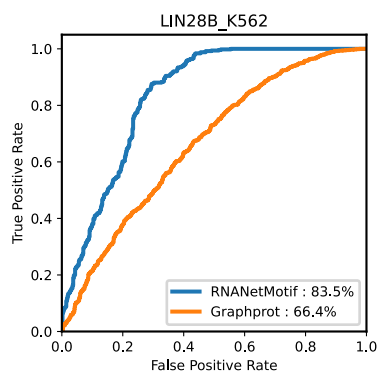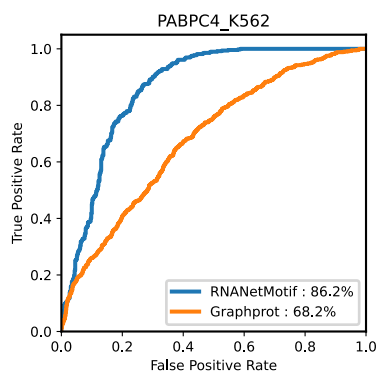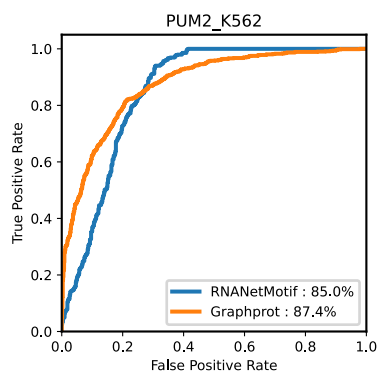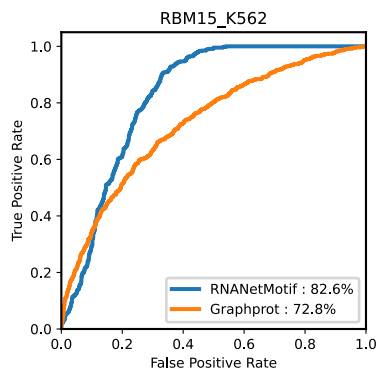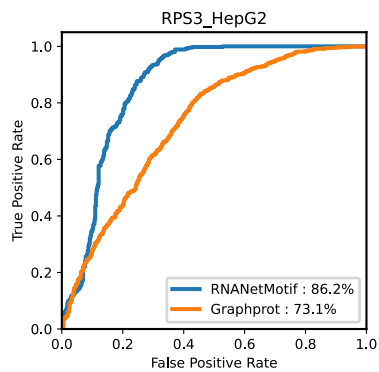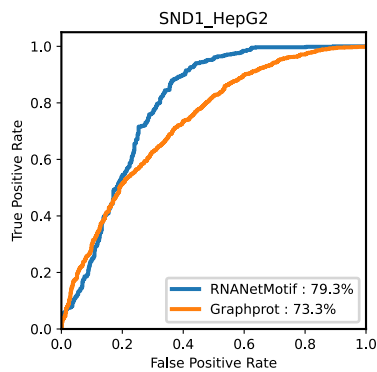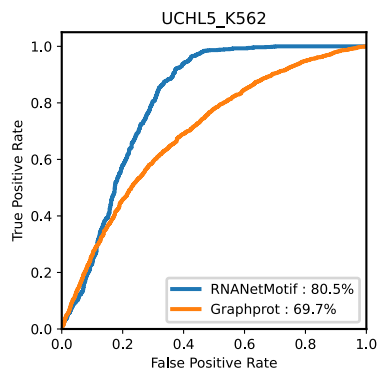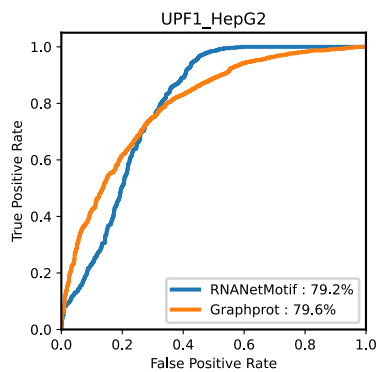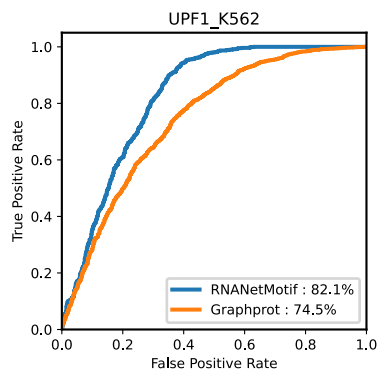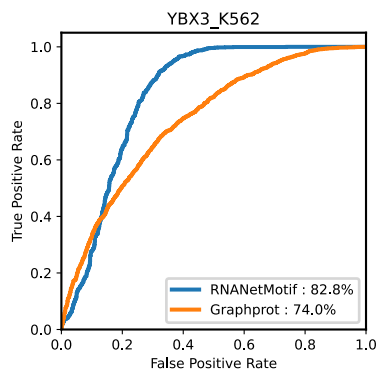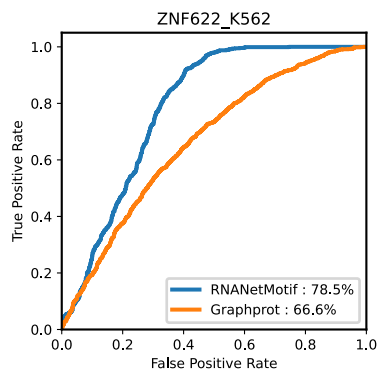

Supplement: S7 Fig — (PDF) [file pcbi.1010293.s010.pdf]

Comparison on running time of different tools

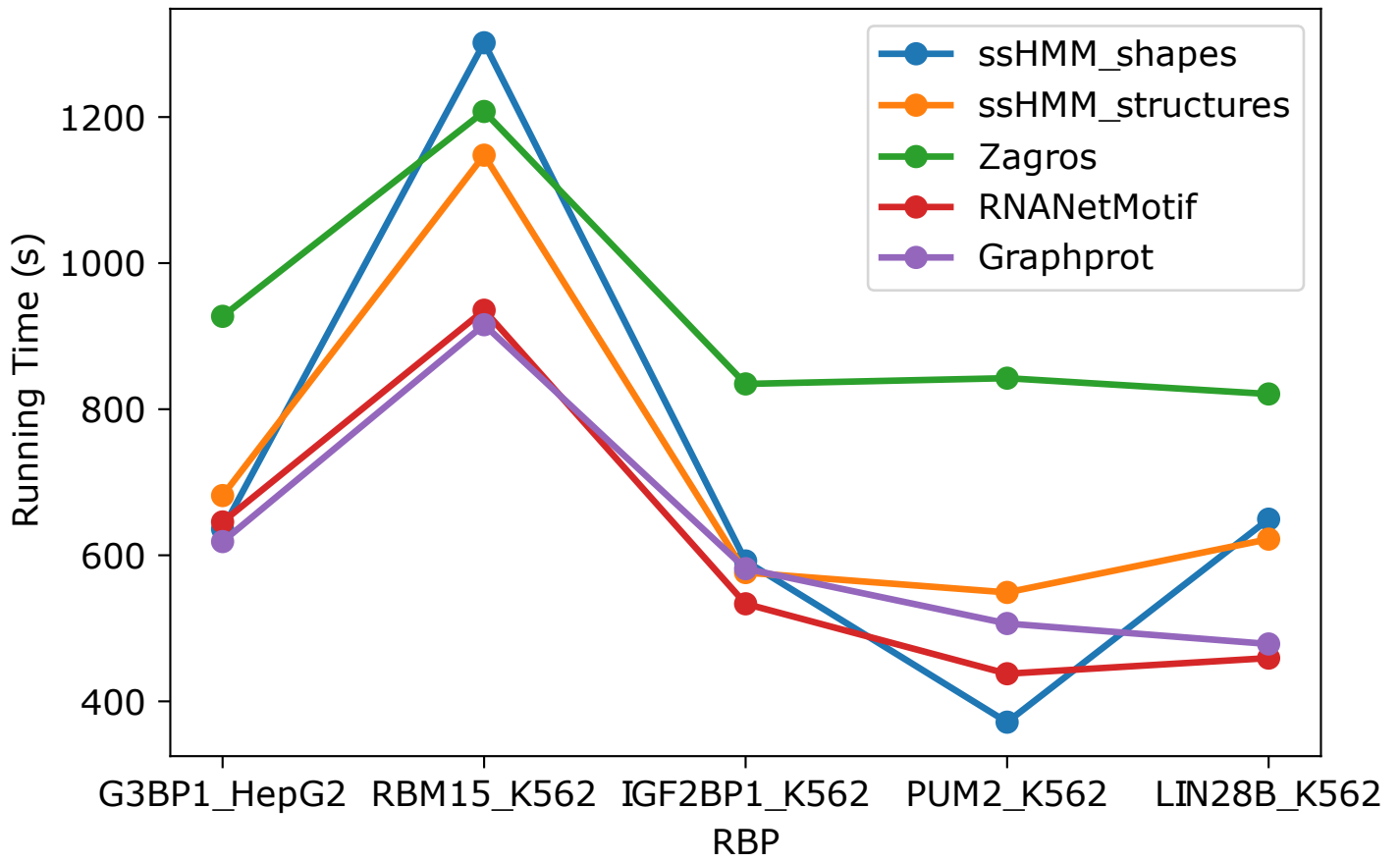

Supplement: S8 Fig — (PDF) [file pcbi.1010293.s011.pdf]

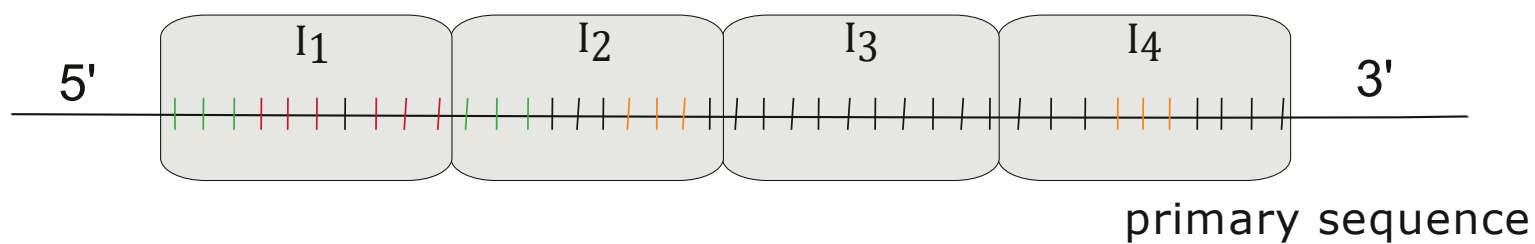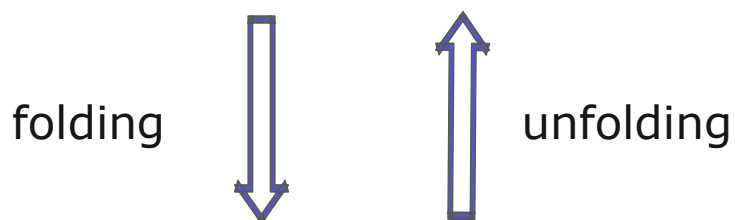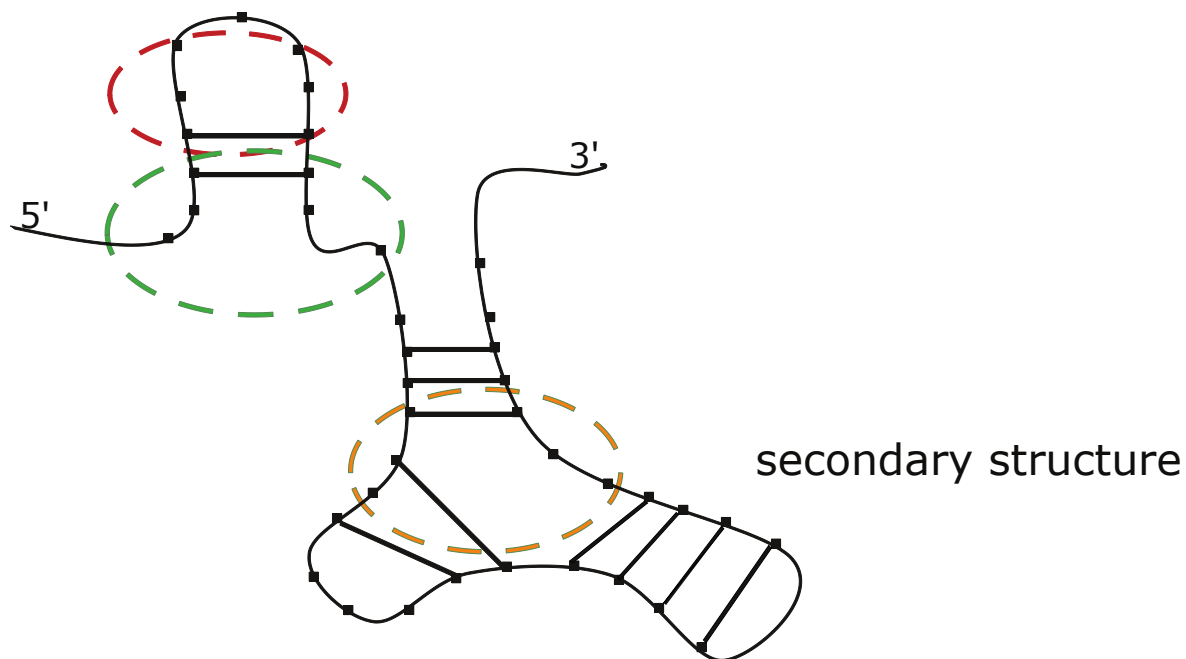

Supplement: S9 Fig — Three EKSes are marked as red, green, and brown dashed circles in the RNA secondary structure. After unfolding, these EKSes are mapped back to primary RNA as gapped sequences with two k-mers falling into four intervals {Ik,k = 1,2,3,4}. (PDF) [file pcbi.1010293.s012.pdf]

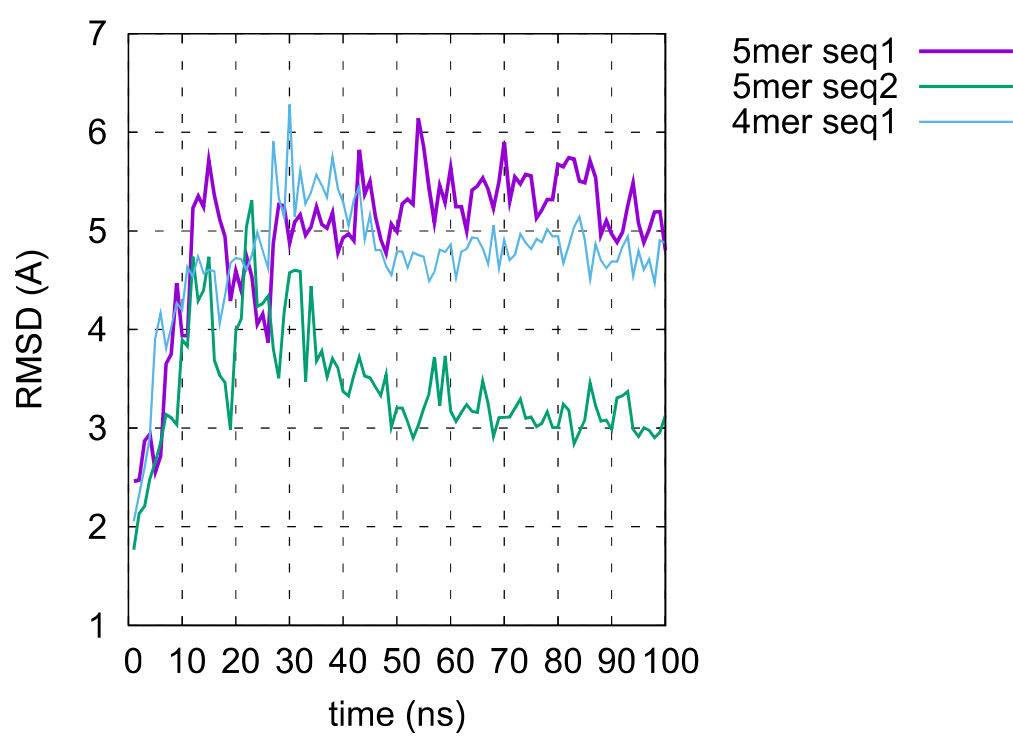

Supplement: S10 Fig — (PDF) [file pcbi.1010293.s013.pdf]

G3BP1\_HepG2

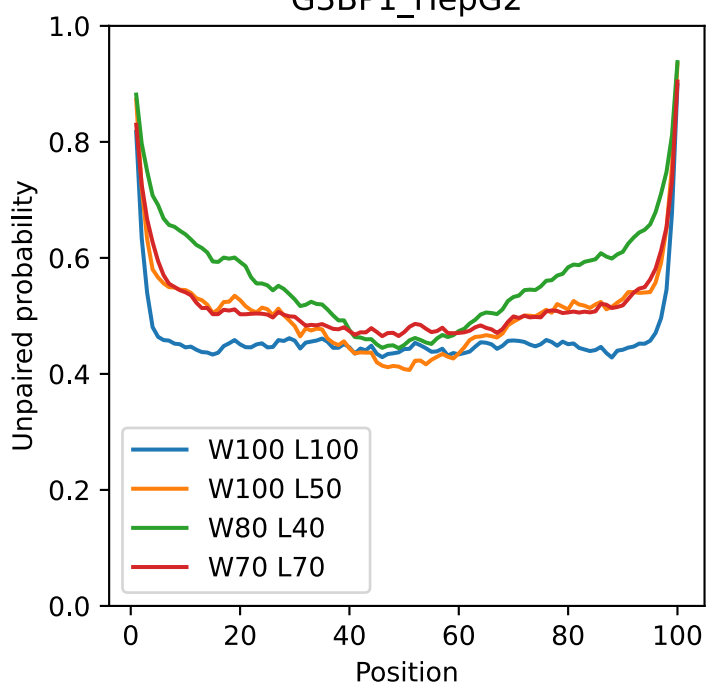

IGF2BP1\_K562

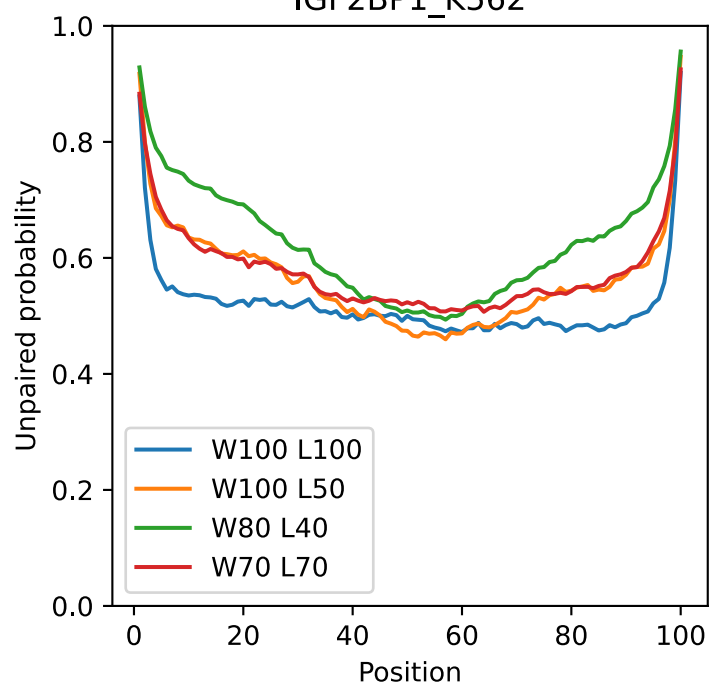

LIN28B\_K562

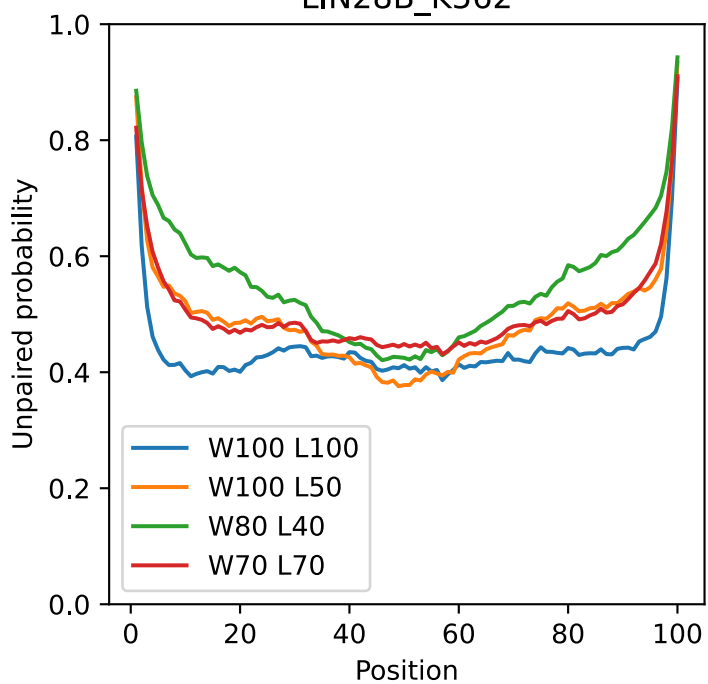

PUM2\_K562

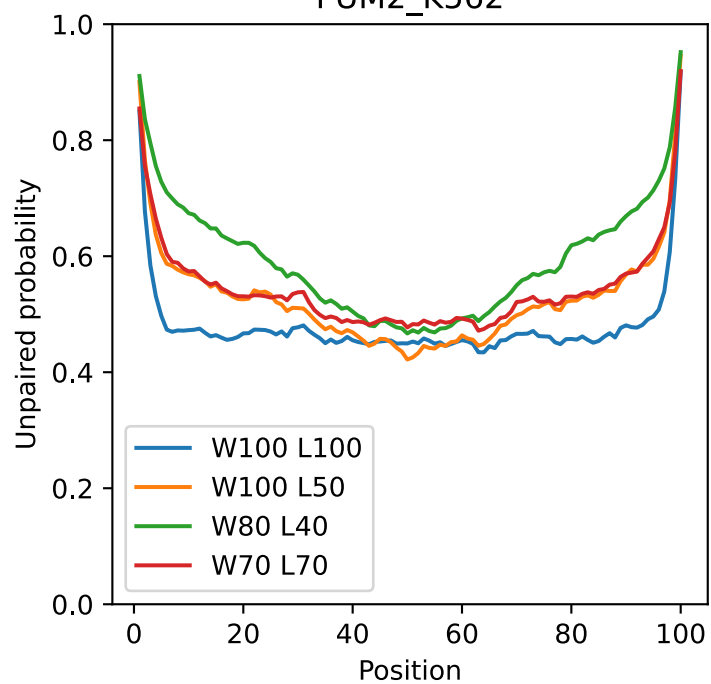

Supplement: S11 Fig — The value of Y axis stands for the trimmed mean of unpaired probability of each position (PDF) [file pcbi.1010293.s014.pdf]

G3BP1\_HepG2

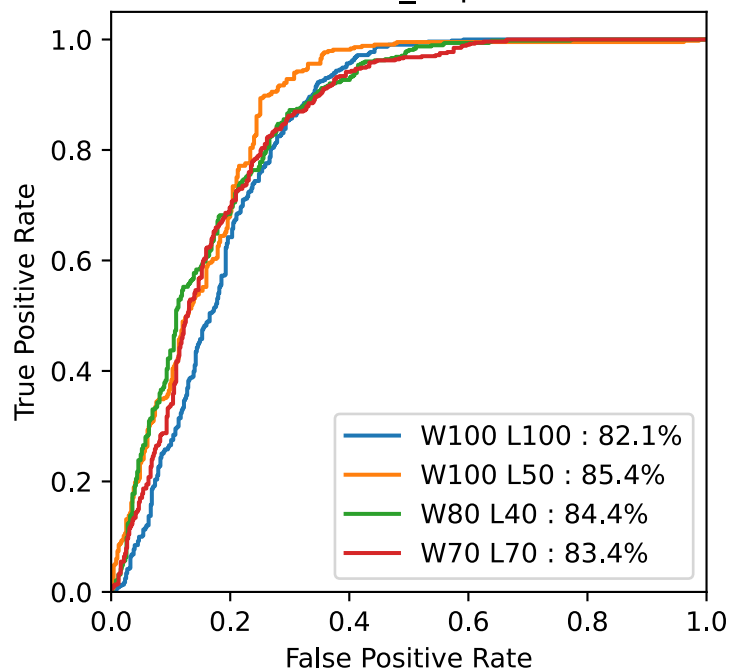

IGF2BP1\_K562

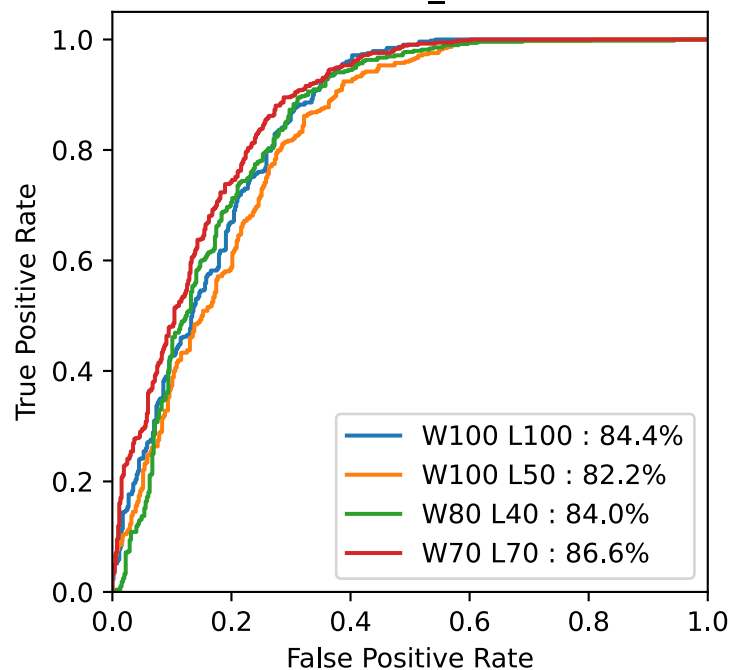

LIN28B\_K562

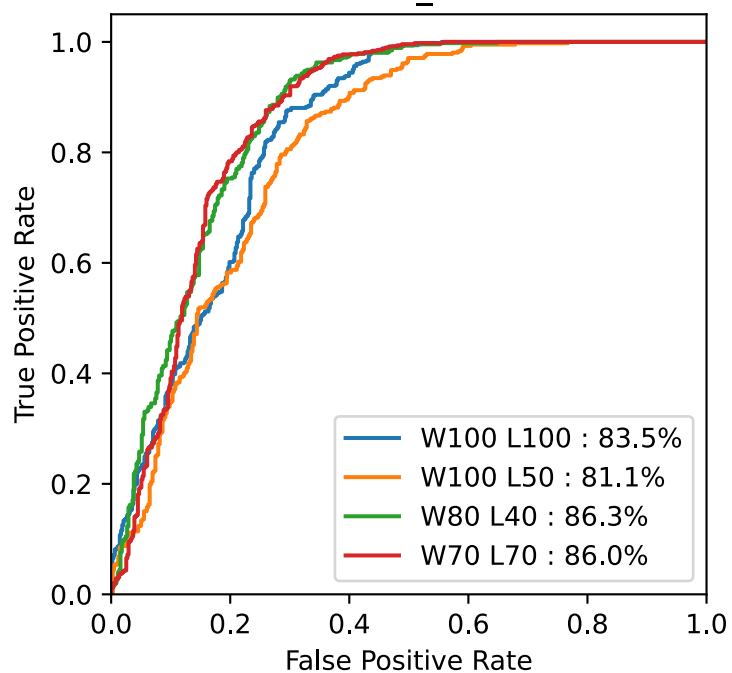

PUM2\_K562

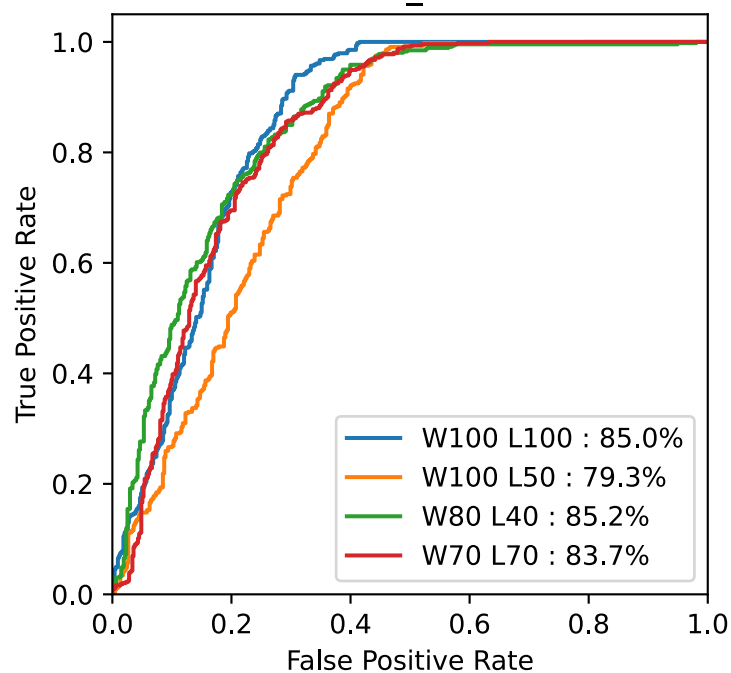

Supplement: S12 Fig — (PDF) [file pcbi.1010293.s015.pdf]

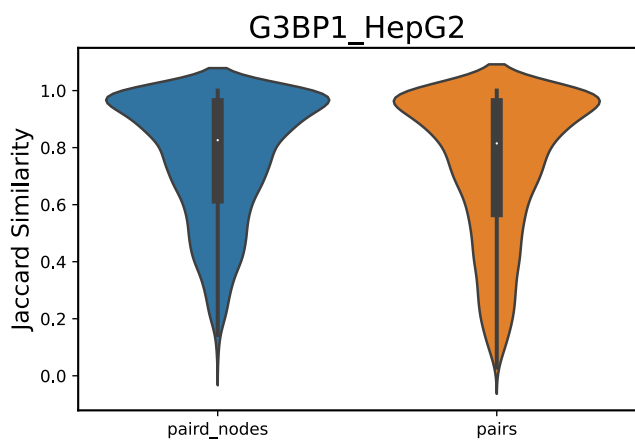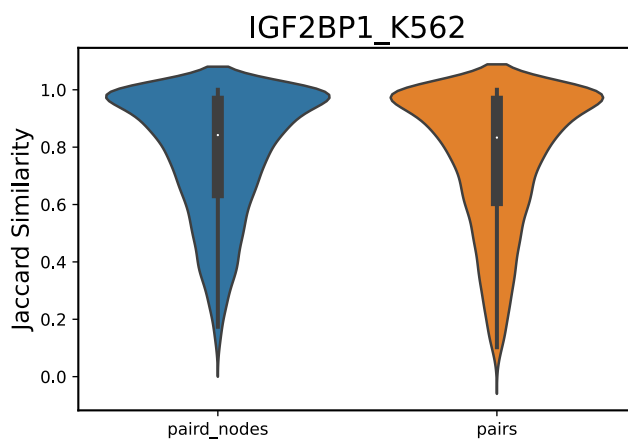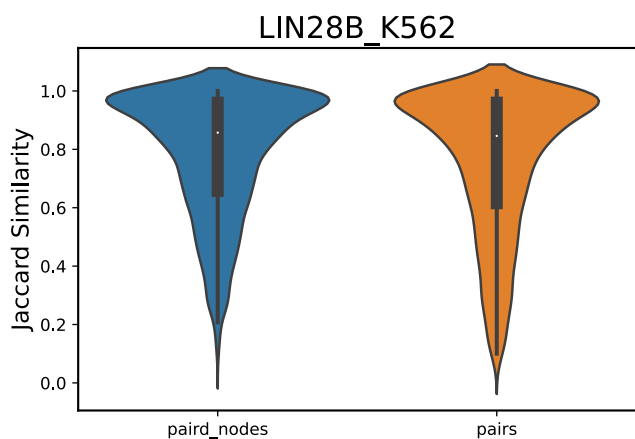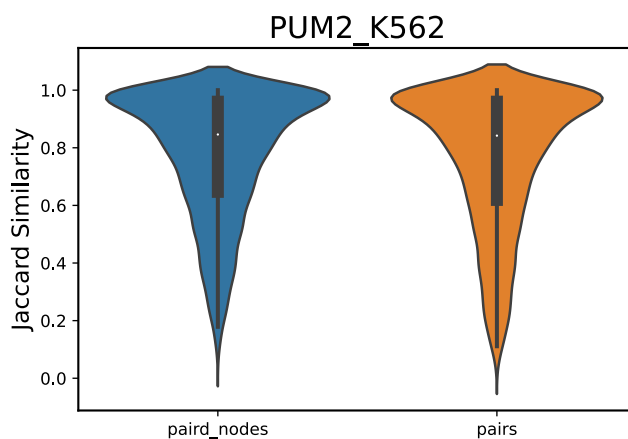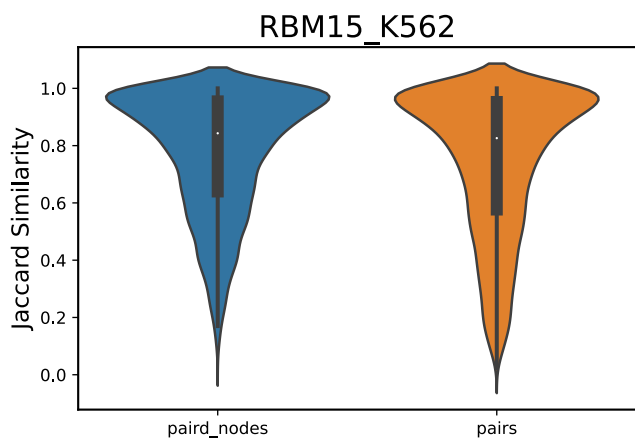

Supplement: S13 Fig — (PDF) [file pcbi.1010293.s016.pdf]
